# Evaluating the cerebrospinal fluid ctDNA detection by next-generation sequencing in the diagnosis of meningeal Carcinomatosis

**DOI:** 10.1186/s12883-019-1554-5

**Published:** 2019-12-19

**Authors:** Yue Zhao, Jun-Ying He, Yue-Li Zou, Xiao-Su Guo, Jun-Zhao Cui, Li Guo, Hui Bu

**Affiliations:** Department of Neurology, The Second Hospital of Hebei Medical University, 215 Heping West Road, Shijiazhuang, 050000 China

**Keywords:** Meningeal Carcinomatosis, Cerebrospinal fluid ctDNA, Next-generation sequencing, Cancer-associated gene mutations

## Abstract

**Background:**

Meningeal carcinomatosis (MC) is the most severe form of brain metastasis and causes significant morbidity and mortality. Currently, the diagnosis of MC is routinely confirmed on the basis of clinical manifestation, positive cerebrospinal fluid (CSF) cytology, and/or neuroimaging features. However, negative rate of CSF cytology and neuroimaging findings often result in a failure to diagnose MC from the patients who actually have the disease. Here we evaluate the CSF circulating tumor DNA (ctDNA) in the diagnosis of MC.

**Methods:**

A total of 35 CSF samples were collected from 35 patients with MC for CSF cytology examination, CSF ctDNA extraction and cancer-associated gene mutations detection by next-generation sequencing (NGS) at the same time.

**Results:**

The most frequent primary tumor in this study was lung cancer (26/35, 74%), followed by gastric cancer (2/35, 6%), breast cancer (2/35, 6%), prostatic cancer (1/35, 3%), parotid gland carcinoma (1/35, 3%) and lymphoma (1/35, 3%) while no primary tumor could be found in the remaining 2 patients in spite of using various inspection methods. Twenty-five CSF samples (25/35; 71%) were found neoplastic cells in CSF cytology examination while all of the 35 CSF samples (35/35; 100%) were revealed having detectable ctDNA in which cancer-associated gene mutations were detected. All of 35 patients with MC in the study underwent contrast-enhanced brain MRI and/or CT and 22 neuroimaging features (22/35; 63%) were consistent with MC. The sensitivity of the neuroimaging was 88% (95% confidence intervals [95% CI], 75 to 100) (*p* = 22/25) and 63% (95% CI, 47 to 79) (*p* = 22/35) compared to those of CSF cytology and CSF ctDNA, respectively. The sensitivity of the CSF cytology was 71% (95% CI, 56 to 86) (*n* = 25/35) compared to that of CSF ctDNA.

**Conclusions:**

This study suggests a higher sensitivity of CSF ctDNA than those of CSF cytology and neuroimaging findings. We find cancer-associated gene mutations in ctDNA from CSF of patients with MC at 100% of our cohort, and utilizing CSF ctDNA as liquid biopsy technology based on the detection of cancer-associated gene mutations may give additional information to diagnose MC with negative CSF cytology and/or negative neuroimaging findings.

## Background

Meningeal carcinomatosis (MC), also called neoplastic meningitis (NM) is the most severe form of brain metastasis and causes significant morbidity and mortality [1]. The early diagnosis and timely treatment are likely to have the greatest impact on improving outcome. However, MC is easily to be missed in diagnosis and easily misdiagnosed because of diverse clinical manifestations and the lack of sensitive and specific diagnostic tools, which has presented difficulties for early treatment of patients with MC [2].

Currently, the diagnosis of MC is routinely confirmed on the basis of clinical signs and symptoms, cerebrospinal fluid (CSF) cytology, and/or neuroimaging (contrast-enhanced brain MRI and/or CT) findings [2]. The presence of neoplastic cells in the CSF is the most useful finding to confirm the diagnosis and CSF cytology remains the gold diagnostic standard, but 25–30% of suspected clinical cases with MC diagnosed based on clinical picture and neuroimaging findings will not be confirmed by this method [3, 4]. Although the positive rate of CSF cytology for neoplastic cells will increase with the number of punctures, the false negative rate is still high, due to the similarity between tumor cells and ependymal cells, contamination by blood brought about by multiple punctures, difficulty of the distinction lymphoma cells caused by viral infections of central nervous system (CNS), few tumor cells in the collected specimen and inadequate preparation of the sample [5]. Measurement of tumor markers in the CSF may be convenient and of value in the adjunctive diagnosis of MC but lack sensitivity and specificity [6–9]. Amplification of tumor specific gene sequences by polymerase chain reaction may be used to detect minimal quantities of neoplastic cells in the CSF, which can be used when the gene mutations in the tumor are known but many cases of MC can be the first presentation of an as yet undetected primary cancer [10]. Contrast-enhanced brain MRI and/or CT is the technique of choice to evaluate patients with suspected MC but still has a 30% incidence of false-negative results [11]. Therefore, it is important to evaluate an alternative method for diagnosis of MC especially for cases with persistently negative CSF cytology and/or persistently negative neuroimaging results.

Circulating tumor DNA (ctDNA) consists of short, double stranded DNA fragments that are released by tumors [12]. The ctDNA implies the entire spectrum of tumor genome aberrations. Current approaches for detection of tumor genome aberrations in ctDNA include PCR-based methods targeting specific mutations and next-generation sequencing (NGS)-based options allowing detection of all possible aberrations in DNA. Improvements in read-length, sequence quality, and throughput allowed NGS to become a more promising method for quantifying ctDNA than PCR-based methods [13]. The tumor-derived ctDNA of plasma has been applied to detect multiple different types of cancer [14, 15]. However, plasma ctDNA from tumors confined to central nervous system was infrequently detectable given that physical obstacles such the blood-brain barrier could prevent ctDNA from entering the blood circulation [14, 16, 17]. Many studies have shown that CSF ctDNA could be an important method of liquid biopsy in patients with CNS cancers [17, 18]. However, only a single tumor type or single primary tumor type was included in these studies. Given that MC is involvement of the leptomeninges by metastatic tumors and can be observed in various kinds of solid tumors, we hope to evaluate the amounts of ctDNA among different primary tumor types and evaluate the clinical value of CSF ctDNA as liquid biopsy medium in the diagnosis of MC. Although the liquid biopsy approach has been shown to be promising, the sensitivity of CSF ctDNA with respect to conventional diagnosis of CSF cytology and neuroimaging findings have not been evaluated in patients with MC. Here, we evaluated the sensitivity of CSF ctDNA with NGS technology as liquid biopsy medium in the diagnosis of patients with MC.

## Methods

### Study subjects

Written informed consent has been obtained from all patients or their legal surrogates. The study protocol has been approved by the Ethics Committee of the Second Hospital of Hebei Medical University, Hebei, China. The patients with MC were enrolled from Department of Neurology in the Second Hospital of Hebei Medical University. In total, 35 patients with MC who underwent lumbar puncture for CSF cytology examination, CSF ctDNA extraction and cancer-associated gene mutations detection by next-generation sequencing (NGS) at the same time and underwent contrast-enhanced brain MRI and/or CT were enrolled in this study between October 2014 and September 2017. The diagnosis of MC had been established by clinical signs and symptoms in addition to positive CSF cytology and/or neuroimaging findings (contrast-enhanced brain MRI or CT) consistent with MC. The clinical signs and symptoms included headache, nausea and vomiting, convulsion, lower back pain, cranial nerve paralysis, paresthesia, gait disturbances, vertigo and defects in mental functioning. The positive CSF cytology result was defined by the morphology of neoplastic cells such as different size with irregular-shape, big nucleus with malignant signs such as lobulated state and malformed buds, increasing chromatin with basophilic coarse particles, mitotic activity with aberrant mitosis, obvious and polymorphic nucleolus accounting for the majority of chromatin and thickening nuclear membrane with saw-tooth-shaped and wear edge. The positive neuroimaging finding was defined as the presence of leptomeningeal enhancement.

### Next-generation sequencing

#### Sample processing

Cerebrospinal fluid samples were collected from each patient. CSF samples in EDTA tubes were centrifuged for 5 min at 1000 g. The pellet was stored at − 20 °C, while the supernatant was centrifuged at 10,000 g for another 30 min [19]. The supernatant was aseptically transferred to pre-labeled Cryotubes stored at − 80 °C. The ctDNA was extracted from at least 5 ml CSF supernatant using the QIAamp Circulating Nucleic Acid kit (QIAGEN) as per the manufacturer’s instructions. Finally, ctDNA was quantified with the Qubit2.1 Fluorometer and Qubit dsDNA HS Assay kit (Life Technologies, Carlsbad, CA) following the recommended protocol.

#### Ion proton library preparation and sequencing

Preparation of the Ion Proton library and DNA sequencing were performed as described in our previous publications [20–22]. For each sample type, an adapter-ligated library was generated with the Ion AmpliSeq Library Kit 2.0 (Life Technologies) according to the manufacturer’s protocol. Briefly, pooled amplicons made from 10~20 ng ctDNA were end-repaired and ligated to Ion Adapters X and P1. AMPure beads (Beckman Coulter, Brea, CA) were used to purify adapter-ligated products, followed by nick-translation and PCR-amplification for a total of 5 cycles. AMPure beads were used to purify the resulting library. And the Agilent 2100 Bioanalyzer and Agilent Bioanalyzer DNA High-Sensitivity LabChip (Agilent Technologies) were used to determine the concentration and size of the library. Sample emulsion PCR and emulsion breaking were performed using the Ion OneTouch™2 system (Life Technologies) with the Ion PI Template OT2 200 Kit v3 (Life Technologies) as per the manufacturer’s instructions. Ion Sphere Particles (ISPs) were recovered, and template-positive ISPs were enriched with Dynabeads MyOne Streptavidin C1 beads (Life Technologies) on the Ion One Touch ES (enrichment system) (Life Technologies). ISPs enrichment was confirmed using the Qubit 2.0 Fluorometer (Life Technologies). The Ion Proton System using Ion PI v2 Chips (Life Technologies) were used to sequence barcoded samples for 100 cycles and the Ion PI Sequencing 200 Kit v3 (Life Technologies) was used for sequencing reactions following the recommended protocol.

We used the SV-OCP143-ctDNA panel (San Valley Biotech Inc., Beijing, China), which is capable of detecting somatic mutations from plasma or tissue samples on 143 cancer-related genes. Since the ctDNA in CSF is comprised of short DNA fragments, amplicons in the panel are specially designed for efficient amplification of ctDNA. For CSF samples, the total read count was more than 25 million to ensure the average base coverage depth over 10,000 x. Additionally, the average uniformity of base coverage was 95.5%. These strict quality control criteria ensured the reliability of sequencing.

#### Variant calling

Initial data from the sequencing runs were processed with the Ion Proton platform-specific pipeline software Torrent Suite v5.0 including generating sequencing reads, trim adapter sequences, filtering and removing poor signal-profile reads as described in our previous publications [20–22]. Initial variant calling from the sequencing data was generated with the TorrentSuite Software with a plug-in ‘variant caller v5.0’. Three filtering steps were used to eliminate erroneous base calling and generate final variant calling. For the first-step, the following filtering criteria were defined for CSF ctDNA: the average total coverage depth>10,000; each variant coverage>10; a variant frequency of each sample>0.1%; and *p* value<0.01. The second-step utilized the Integrative Genomics Viewer (IGV) software (http//www.broadinstitute.org/igv) or Samtools software (http://samtools.sourceforge.net) to eliminate possible DNA strand-specific errors after visual examination of called mutations.

## Results

### Characteristics of patients with MC

The clinical characteristics of 35 patients with MC were summarized in Table 1. There were 16 male and 19 female patients. The date of MC diagnosis was defined as the date of the first positive cytological study or of the first positive neuroimaging findings consistent with MC. The median age at the time of diagnosis of MC was 52 (range, 23–70) years. In 33 of 35 cases, the primary tumors were determined while no primary tumor could be found in the remaining 2 patients in spite of using various inspection methods. Of 33 patients who had clear and definite primary tumor, primary tumor included lung cancer (26 cases), gastric cancer (2 cases), breast cancer (2 cases), prostatic cancer (1 case), parotid carcinoma (1 case), and lymphoma (1 case). Of the 26 patients with lung cancer, a large majority of the patients were found to have adenocarcinoma histology (21/26, 81%). Of 33 patients who had clear and definite primary tumor, 7 patients (7/33, 21%) were diagnosed as MC before the initial presentation of primary tumor. The median time of the remaining 26 patients with MC from the initial diagnosis of the primary tumor to the diagnosis of MC was 355 (range, 1–2566) days. Headache, nausea and vomiting were the most common The baseline Karnofsky Performance Scores (KPS) was 60~80 in the majority of patients (30/35, 86%).

**Table 1:** Clinical features of patients with MC

No.	Primary tumor	Clinical manifestation	KPS
N01	Unknown	Headache, nausea and vomiting	60
N02	Breast cancer	Headache, nausea and vomiting	60
N03	Unknown	Nausea and blurred vision	30
N04	Gastric cancer	Headache, dizziness and paralysis	80
N05	Gastric cancer	Headache and nausea	70
N06	Lymphoma	Headache	70
N07	Prostatic cancer	Headache	70
N08	Parotid carcinoma	Headache, nausea and vomiting	80
N09	Lung cancer	Headache, nausea and vomiting	70
N10	Lung cancer	Headache and decreased vision	80
N11	Lung cancer	Headache, nausea and vomiting	80
N12	Lung cancer	Headache, nausea and vomiting	70
N13	Lung cancer	Headache and dizziness	70
N14	Lung cancer	Headache, nausea, vomiting and back pain	70
N15	Lung cancer	Headache and neck pain	70
N16	Lung cancer	Headache, nausea and vomiting	60
N17	Lung cancer	Headache and dysarthria	40
N18	Lung cancer	Headache, nausea and vomiting	80
N19	Lung cancer	Leg pain	70
N20	Lung cancer	Headache, nausea, vomiting, dizziness and neck pain	70
N21	Lung cancer	Headache, nausea, vomiting and back pain	60
N22	Lung cancer	Headache, nausea, vomiting and dizziness	60
N23	Lung cancer	Headache, nausea, vomiting, dizziness and neck pain	40
N24	Lung cancer	Headache, nausea, vomiting, dizziness and hearing loss	60
N25	Lung cancer	Headache, nausea, vomiting and dizziness	70
N26	Lung cancer	Headache, nausea, vomiting, confusion and decreased vision	30
N27	Lung cancer	Blurred vision	70
N28	Lung cancer	Headache and dizziness	50
N29	Lung cancer	Headache, nausea, vomiting and back pain	70
N30	Lung cancer	Headache, nausea, vomiting, confusion and back pain	70
N31	Lung cancer	Headache, nausea, vomiting, and diplopia	70
N32	Lung cancer	Headache, nausea and vomiting	70
N33	Breast cancer	Headache and paralysis	80
N34	Lung cancer	Headache, nausea and vomiting	60
N35	Lung cancer	Speech disorder	80

### Sensitivity of all methods analyzed

The sensitivity of all methods analyzed was presented in Table 3. A total of 35 CSF samples were collected from 35 patients with MC for CSF cytology examination, CSF ctDNA extraction and cancer-associated gene mutations detection by NGS at the same time. Twenty-five CSF samples (25/35; 71%) were CSF cytology positive while all of the 35 CSF samples (35/35; 100%) were revealed having detectable tumor-derived ctDNA in which cancer-associated gene mutations were detected by NGS. The CSF immunocytochemistry investigations were performed in 14 CSF specimens with positive CSF cytology. All of 35 patients with MC in the study underwent contrast-enhanced brain MRI and/or CT and 22 neuroimaging features (22/35; 63%) were consistent with MC, while of 22 patients with leptomeningeal enhancement, 5 patients combined parenchymal brain metastasis. (Table 2 and Table 3).

**Table 2:** Concurrent result of CSF cytology, CSF ctDNA and neuroimaging findings in patients with MC

No.	CSF immunocytochemistry	Neuroimaging findings	Concurrent result of CSF cytology	Concentration of CSF ctDNA (ng/ul)	CSF ctDNA gene mutations (mutation frequency)	Parenchymal brain metastasis
N01	CK7(+), Ki-67(+), ER(−), PR(−)	Leptomeningeal enhancement	+	5.36	TP53 R196* (68.55%) VHL R200W (5.19%)	_
N02	Ki-67(+), CK7(+), ER(−), FR(−)	Leptomeningeal enhancement	+	0.512	PTEN K13Q (0.455%) NOTCH1 (38.86%)	_
N03	Ki-67(+), CEA(−), CK20(−), CK7(−), CK20(−)	Leptomeningeal enhancement	+	0.204	PTEN K13Q (0.311%)	+
N04	Ki-67(+), CEA(+)	Leptomeningeal enhancement	+	2.08	TP53 L194R (45.69%)	_
N05	Not available	Leptomeningeal enhancement	_	1.17	TP53R248Q (1.464%)	_
N06	Not available	Leptomeningeal enhancement	_	1.36	PTEN R14G (0.204%)	_
N07	Not available	Leptomeningeal enhancement	_	0.29	TP53 P301Q (0.761%)	_
N08	CK7 (+)	Leptomeningeal enhancement	+	6.82	TP53 C242G (72.914%)	_
N09	NapsinA (+), CK7(+)	Negative	+	0.144	TP53 I195F (89.921%)	_
N10	Not available	Negative	+	0.238	TP53 F113 V (55.412%)	_
N11	NapsinA (+), TTF1(+), CK7(+)	Negative	+	5.36	TP53 R337C (53.924%)	_
N12	CK7(+), NapsinA(+), Ki-67(−), TTF1(−), CDX2(−), CD20(−)	Leptomeningeal enhancement	+	1.74	EGFR L858R (82.178%)	+
N13	TTF1(+), NapsinA (+)	Negative	+	3.46	EGFR L858R (29.903%)	_
N14	Not available	Leptomeningeal enhancement	_	3.52	EGFR L858R (33.003%)	_
N15	Not available	Negative	+	3.08	EGFR L858R (14.159%)	_
N16	Not available	Negative	_	25.6	EGFR L858R (0.229%)	_
N17	Not available	Leptomeningeal enhancement	+	1.65	TP53 P301Q (0.482%)	_
N18	Not available	Negative	+	2.26	EGFR E746_A750del (38.178%)	_
N19	Not available	Leptomeningeal enhancement	_	30.6	EGFR T790 M (22.61%)	_
N20	Not available	Leptomeningeal enhancement	+	2.58	TP53 G245S (0.497%)	_
N21	Not available	Negative	+	26.6	EGFR L858R (78.401%)	_
N22	NapsinA(+), TTF1(−), Ki-67(−), CEA(+)	Negative	+	1.42	EGFR L858R (12.359%)	_
N23	Not available	Negative	+	2.76	EGFR L858R (24.468%)	_
N24	Not available	Negative	+	3.58	EGFR L858R (5.535%)	_
N25	NapsinA(+)	Leptomeningeal enhancement	+	2.92	PTEN C136Y (1.431%)	_
N26	Not available	Leptomeningeal enhancement	+	7.54	EGFR T790 M (6.286%)	_
N27	CK7(−), Ki-67(−), TTF1(−), NapsinA(−), CK20(−), CDX20(−), CDX2(−), CEA(−), FAP(−)	Leptomeningeal enhancement	+	1.23	EGFR L858R (79.739%)	+
N28	Not available	Leptomeningeal enhancement	–	4.1	EGFR L858R (1.606%)	+
N29	Not available	Negative	+	3.36	PTEN K13Q (0.387%)	_
N30	Not available	Negative	+	4.16	EGFR L858R (24.22%)	_
N31	CK7(+), NapsinA(+), Ki-67(−), PR(−), TTF1(−)	Leptomeningeal enhancement	+	4.04	EGFR T790 M (5.233%)	_
N32	Ki-67(−), CEA(+), CK7(+), TTF1(+), CDX2(−), CK20(−), NapsinA(+), ER(−), PR(−)	Leptomeningeal enhancement	+	3.8	EGFR L858R (3.74%)	_
N33	Not available	Leptomeningeal enhancement	_	1.51	PTEN K13Q (0.464%)	+
N34	Not available	Leptomeningeal enhancement	_	1.09	TP 53 P250L (84.24%)	_
N35	Not available	Leptomeningeal enhancement	_	1.42	FLT3 Y842C (0.404%)	_

**Table 3:** Sensitivity of all methods analyzed

	% Sensitivity (no. of positive patients) of each method		
	CSF cytology	Neuroimaging	ctDNA
Patients (35)	71.43 (25)	62.86 (22)	100 (35)

The sensitivity of the neuroimaging was 88% (95% confidence intervals [95% CI], 75 to 100) (*p* = 22/25) and 63% (95% CI, 47 to 79) (p = 22/35) compared to those of CSF cytology and CSF ctDNA, respectively. The sensitivity of the CSF cytology was 71% (95% CI, 56 to 86) (*n* = 25/35) compared to that of CSF ctDNA. The specificity of CSF ctDNA in all cases was 100%. These data suggest a higher sensitivity of CSF ctDNA than those of CSF cytology and neuroimaging findings.

**Fig. 1:**
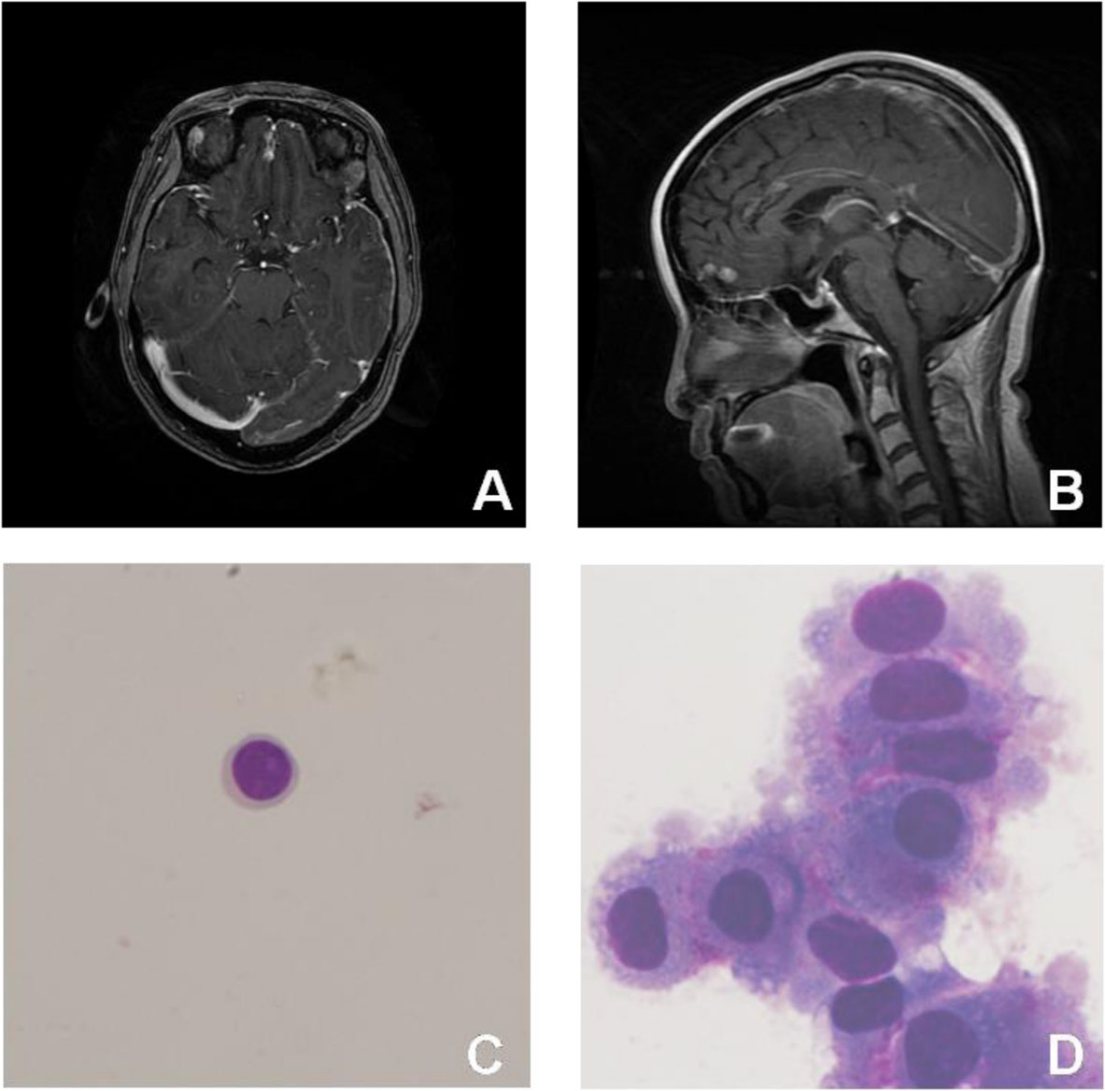
Head contrast enhanced MRI (**a** and **b**) of one patient showed abnormally uniform leptomeningeal enhancement. At the same time, no neoplastic cells were found in CSF by CSF cytology (**c**) while CSF sample revealed ctDNA and showed cancer-associated gene mutations of PTEN K13Q (0.464%), TP53 P301Q (0.809%), VHL R161Q (0.213%), CDKN2A A68T(0.235%) and FGFR2 Y375C (0.207%) by NGS. CSF cytology (**d**) 1 year later showed a large number of neoplastic cells

Nine cases were CSF cytology negative but neuroimaging positive while CSF ctDNA with cancer-associated gene mutations could be detected in them. Interestingly, neoplastic cells were found in CSF of patient N33 after 1 year from the first lumber puncture (Fig. 1).

### Concordance of EGFR activating mutations in primary lung adenocarcinomas and MC CSF samples

The comparisons between primary tumors and MC CSF samples were available only in 6 MC patients with lung adenocarcinoma (Table 4). We collected CSF samples from N10, N11, N15 and N31 during the tyrosine kinase inhibitor (TKI) therapy and N16 and N24 samples several months after the TKI. All patients, as carriers of EGFR mutations, of which 2 patients were found to contain exon 19 deletion and 4 patients to contain L858R. Additionally, CSF sample N11 also exhibited T790 M and E709A while CSF sample N31 also contained T790 M.

**Table 4:** The comparisons of EGFR activating mutations between primary lung adenocarcinomas and MC CSF samples

CSF sample No.	Primary lung adenocarcinoma tissues	CSF samples
N10	EGFR 19Del	EGFR 19Del
N11	EGFR L858R	EGFR L858R, E709A, T790 M
N15	EGFR L858R	EGFR L858R
N16	EGFR L858R	EGFR L858R
N24	EGFR L858R	EGFR L858R
N31	EGFR 19Del	EGFR 19Del, T790 M

## Discussion

In the present study, we compared the sensitivity of three methods including CSF cytology, neuroimaging findings and CSF ctDNA in the diagnosis of MC. The study revealed that CSF ctDNA had a higher sensitivity than the CSF cytology and neuroimaging findings. Most studies of ctDNA published to date have demonstrated the importance of plasma ctDNA as a liquid biopsy medium for various kinds of cancer given that benign tumors and non-neoplastic conditions do not generally give rise to ctDNA [23]. Similarly, CSF ctDNA may be useful in complementing the diagnosis of MC especially for cases with persistently negative CSF cytology and/or negative neuroimaging findings.

Currently, the presence of neoplastic cells in the CSF is the most useful criteria to confirm the diagnosis of MC and CSF cytology remains the gold diagnostic standard although the false negative rate of CSF cytology is still high. In our study, we found the sensitivity of CSF ctDNA is higher than that of CSF cytology. Previous study [14] had presented that ctDNA was often present in patients without detectable circulating tumor cells. Contrast-enhanced brain MRI is the technique of choice to evaluate patients with suspected MC but still has a 30% incidence of false-negative results [11]. The sensitivity of the neuroimaging was 63% compared to that of CSF-derived ctDNA by next-generation sequencing in our study. Neuroimaging studies are often noninformative or slow to reflect progression. Repeated neuroimaging also subjects patients to radiation, whereas monitoring ctDNA is noninvasive. However, other studies demonstrate that a cancer containing ~ 50 million malignant (rather than benign) cells releases sufficient DNA for detection in the circulation [24]. A cancer of this size is well below that required for definitive imaging at present. Measurement of tumor markers in the CSF may be convenient and of value in the adjunctive diagnosis of MC but lack sensitivity and specificity [6–9]. Unlike tumor markers such as CA19–9 or CEA, which are expressed in normal cells as well as in neoplastic cells, gene mutations of a clonal nature are only found in neoplasms.

In cancer patients, ctDNA are thought to be released in plasma as a result of tumor cells apoptosis and/or necrosis [14, 25]. ctDNA has played an important role in monitoring disease status of advanced cancer patients as a promising blood-based biomarker [26]. Many studies showed that ctDNA analysis can be utilized in early diagnosis of human malignancies including pancreatic, renal, advanced ovarian, colorectal, bladder, gastroesophageal, breast, melanoma, hepatocellular, head and neck cancers [14, 19, 27–29]. Previous study [14] showed that less than 10% of patients with gliomas harbored detectable ctDNA in the plasma while Yuxuan Wang et al. [18] demonstrated they identified detectable levels of CSF ctDNA in 74% of patients with primary tumors of the brain or spinal cord whereas no ctDNA was detected in patients whose tumors were not directly adjacent to a CSF reservoir. We attribute the 100% detection rate of CSF-derived ctDNA in our study to the reason that MC disseminates over the leptomeningeal surface, neoplastic cells shed into CSF and CSF is in direct contact with neoplastic cells and meninges while owing to physical obstacles such the blood–brain barrier, CSF ctDNA is unable to circulate fully within the blood system, resulting in a limited amount of ctDNA from CNS being released to plasma circulation.

Elena et al. [19] used next-generation sequencing to reveal somatic alterations in tumor-derived DNA from CSF in patients with CNS metastases mainly brain parenchyma metastasis while the positive rate is not 100 %. Our study showed a good result due to the patients who were recruited in this study were all definite MC cases. Tumor cells of MC cases were circulating in the CSF, which led to be easier to be caught. Consequently, this technique is more suitable for the diagnosis of MC than brain parenchyma metastasis.

As per the analysis for concordance of EGFR activating mutations between primary lung adenocarcinomas and MC, on the basis of patients’ CSF samples, the results showed that, EGFR activating mutations in CSF samples were consistent with those in primary adenocarcinomas. The showing of T790 M in two CSF samples, is possibly attributed to the fact that both two CSF samples were sequentially collected during the process of TKI therapy.

Our study find DNA mutation in CSF of patients with MC at 100% of our cohort, and it may give additional information to diagnose MC with negative CSF cytology. Furthermore, the type of tumor-associated gene mutations can provide many clues to the primary tumor type especially for patients whose primary tumor wouldn’t be found in spite of using various inspection methods. Additionally, looking for and knowing the primary tumor is the rather important diagnostic dependency.

However, our current study does have some limitations; for example, the cohort has had relative small numbers of patients and we used a smaller 143 gene panel. Though it was the most comprehensive gene panel at that time, including hot-spot mutation, copy number variants, gene fusion, and gene therapy information, it could not detect all possible aberrations in DNA. Thus, future study with larger size of samples from other departments and multiple institutions and using larger gene panel could help us to solve these issues.

## Conclusions

This study suggests a higher sensitivity of CSF ctDNA than those of CSF cytology and neuroimaging findings. We find cancer-associated gene mutations in ctDNA from CSF of patients with MC at 100% of our cohort, and utilizing CSF ctDNA as liquid biopsy technology based on the detection of cancer-associated gene mutations may give additional information to diagnose MC with negative CSF cytology and/or negative neuroimaging findings. At the same time, cancer-associated gene mutations provide a reliable theory for MC targeted therapy.

## Data Availability

Study data are available from the corresponding author upon reasonable request.

## Abbreviations

CNS: Central nervous system
CSF: Cerebrospinal fluid
CT: Computed tomography
ctDNA: Circulating tumor DNA
KPS: Karnofsky Performance Scores
MC: Meningeal carcinomatosis
MRI: Magnetic resonance imaging
NGS: Next-generation sequencing
NM: Neoplastic meningitis
TKI: Tyrosine kinase inhibitor
